# Mobilizable shuttle vectors with fluorescent markers functional across different species of bacteria

**DOI:** 10.1128/aem.00045-25

**Published:** 2025-05-12

**Authors:** Zackary R. Armstrong, Janie Alonso, Venus Stanton, Nikhil Patel, Xhavit Zogaj, Sebastian S. Cocioba, Karl E. Klose

**Affiliations:** 1 South Texas Center for Emerging Infectious Diseases and Department of Molecular Microbiology and Immunology, University of Texas San Antonio 250767 , San Antonio, Texas, USA; 2 Binomica Labs, Long Island City, New York, USA; Indiana University Bloomington, Bloomington, Indiana, USA

**Keywords:** plasmid, non-model bacteria, chromoproteins, Golden Gate assembly, *Francisella novicida*, *Acinetobacter baumannii*, *Vibrio alginolyticus*, promoter exchange, RSF1010, broad-host range

## Abstract

Chromophore-containing proteins (CCPs), including fluorescent and non-fluorescent (chromoproteins), have been widely used in microbiological research. However, several roadblocks often limit their use in non-model bacterial species, including efficient transformation, suitable plasmid origins of replication, and optimal promoter choice. Here, we have engineered a set of 32 shuttle plasmids designed to overcome these roadblocks in an effort to streamline this process for future research. We have selected eight different CCPs: eforCP, YukonOFP, DasherGFP, tinsel Purple, aeBlue, FuGFP, super-folder GFP, and super-folder Cherry2. To broaden the potential host range, we utilized two distinct backbones with p15a either fused to a *Francisella* origin (FnOri) or to the broad host origin RSF1010 and included a transfer origin (*oriT*) to facilitate transformation via conjugation. Moreover, we have created versions of each vector, which confer resistance to either kanamycin or chloramphenicol. Lastly, to enable promoter-swapping, we engineered the constitutive p*J23100* promoter element to be flanked by *BsaI* sites, thereby enabling promoter exchange by the Golden Gate assembly to evaluate CCP expression with different host promoters. To demonstrate the usability of the pKEK-Chrom plasmid series, we evaluated their expression in *Escherichia coli*, *Shewanella oneidensis*, and *Vibrio alginolyticus*. We further demonstrated the utility of promoter swapping in *Francisella novicida* and validated the functionality of the RSF1010 origin in *Acinetobacter baumannii*. In summary, the pKEK-Chrom plasmid series provides a palette of different CCPs that streamline their use in non-model gram-negative bacteria.

**IMPORTANCE:**

Chromophore-containing proteins (CCPs), including both fluorescent proteins and pigment-producing (non-fluorescent) chromoproteins, have become invaluable tools for microbial research. However, their successful implementation in understudied bacterial species lacking established genetic tools often requires substantial time and resources. Our goal was to develop a set of plasmid-based vectors that could streamline CCP expression in gram-negative bacteria. To do so, we developed a set of 32 plasmid vectors, the pKEK-Chrom plasmid series, specifically designed to facilitate CCP expression across different bacteria, including *Escherichia coli*, *Vibrio alginolyticus*, *Shewanella oneidensis*, *Francisella novicida*, and *Acinetobacter baumannii*.

## INTRODUCTION

Chromophore-containing proteins (CCPs), including fluorescent proteins (FPs) and non-fluorescent chromoproteins (CPs), have become invaluable tools in modern microbiology research (molecular reporters, genetic complementation, protein localization, *in vivo* imaging, and biosensors) (1). Since the discovery of green fluorescent protein (GFP) from the jellyfish *Aequorea victoria* (2), a palette of GFP-like FPs with distinct chromophores (fluorescent and color moieties) with enhanced unique spectral and biophysical properties has been characterized, including eforCP/RFP (3, 4), DasherGFP (5), Free-use GFP (FuGFP) (6), tinsel Purple (tsPurple) (4), aeBlue (4, 7), YukonOFP (8), super-folder GFP (sfGFP) (9), and super-folder Cherry (sfCherry) (10). Because both fluorescent and non-fluorescent (chromoproteins) proteins are composed of chromophores that ultimately define their spectral properties (e.g., fluorescence and non-fluorescence), we adopt the term chromophore-containing proteins (CCPs) as a generalized descriptor throughout this work. The variety of spectral properties provides researchers with CCPs that can be tailored to specific experimental needs (Table 1).

**TABLE 1:** Properties of CCPs

Protein	Accession	Origin	Absorbance (nm)		Reference(s)
			Exc	Em	
FuGFP	7BLNH	*Aequorea Victoria;* modified (Nick Coleman and Mark Somerville)	395	503	(6)
sfGFP	B4SOW	*Aequorea Victoria;* modified	488	510	(9)
DasherGFP	QSX72528.1	*Obelia* sp.; modified (ATUM Protein Paintbox)	505	525	(5, 11, 12)
YukonOFP	AAZ14789 ^*a*^ [Table-fn T1_FN1]	*Corynactis californica*, modified	440	565	(8, 11, 12)
EforCP/RFP	TDA9A	*Echinopara forskaliana*	589	609	(3, 4)
sfCherry2	UHMHB	*Discosoma* sp.*; modified*	580	610	(10)
aeCP597 (Blue) chromoprotein	LFOL4	*Actinia equina*	NF	NF	(4, 7)
tsPurple chromoprotein	N6AXC	*Actinia equina*; modified	NF	NF	(4)

^*a*^ Not listed in the fluorescent protein database (FPBase ID).

For microbiological applications, these CCPs are typically expressed from plasmid-based vectors that contain appropriate promoters and origins of replication (Ori). These elements ensure sufficient expression of the target proteins and maintenance of the expression vector within the host species. However, using these proteins in non-model bacterial species presents challenges, including the need for established transformation methods, organism-specific promoter activity, and suitable origins of replication. Overcoming these challenges is essential for broadening the application of CCPs in non-model organisms that lack established genetic systems. Therefore, developing a versatile set of plasmid vectors capable of expressing various CCPs would streamline their use in diverse bacterial species. Vectors would ideally be easily adapted to include organism-specific components, such as promoters and Ori, allowing researchers to apply these powerful tools more widely in non-model bacteria.

Gram-negative bacteria, such as *Escherichia coli*, *Vibrio* spp., *Francisella* spp., *Shewanella oneidensi*s, and *Acinetobacter baumannii*, have distinct organism-specific requirements for growth, stable plasmid maintenance, and expression. While *Vibrio* and *Shewanella* spp. are more closely related to *E. coli*, allowing a stable maintenance of most *E. coli*-optimized promoters and plasmid origins of replication (e.g., p15a), their differing metabolic and growth requirements (e.g., halophilic media, growth at 30°C) may affect CCP expression and activity. In contrast, *Francisella* spp., which are facultative intracellular bacteria and more distantly related to *E. coli*, face two major roadblocks: (i) *E. coli*-derived plasmid origins of replication are not stably maintained within *Francisella* spp., and (ii) their transcription machinery, including two unique RNA polymerase α subunits, fails to drive transcription from *E. coli*-optimized promoters (13, 14). The identification of the cryptic plasmid pFNL10 in a *F. novicida*-like species paved the way for the first shuttle vectors for *Francisella* research (15–17). Over the last decade, additional *Francisella* promoters (e.g., pFTN_1451 and p*groEL*) have been identified, leading to the development of a broader range of genetic tools for understanding *Francisella* pathogenesis (13, 14, 18–20). *A. baumannii* is an aerobic, non-motile, opportunistic pathogen that has garnered attention due to the prevalence of multi-drug-resistant strains, resulting in its classification as an ESKAPE pathogen (21, 22). *A. baumannii* recognizes *E. coli*-optimized promoter elements, but the stable maintenance of shuttle vectors requires specific origins of replication. Over the past 30 years, the *A. calcoaceticus* lwoffii cryptic plasmid-derived origin and the IncQ (RSF1010) broad-host range origin have been shown to be stably maintained within *A. baumannii* (23, 24). These various factors highlight the challenges of developing genetic tools for non-model organisms.

Here, we constructed a set of 32 mobilizable shuttle vectors for the expression of eight different CCPs in diverse gram-negative bacteria. We utilized two distinct origins of replication: p15a fused to a *Francisella*-specific origin (p15a-FnOri) or RSF1010-p15a, facilitating plasmid maintenance across different host bacteria. Additionally, we incorporated a constitutive *E. coli*-optimized promoter flanked by *BsaI* restriction sites to enable promoter swapping via the Golden Gate assembly. The plasmids also contain transfer origins (*oriT*) to allow their introduction into recipient bacteria by conjugation. To further broaden the applicability of this plasmid set, we constructed versions containing selectable markers conferring resistance to either kanamycin or chloramphenicol. We evaluated plasmids for CCP expression in *E. coli*, *V. alginolyticus*, and *S. oneidensis* and demonstrated the feasibility of using these plasmids to mediate promoter swapping and CCP expression in *F. novicida*, as well as in *A. baumannii*.

## MATERIALS AND METHODS

### Bacterial strains and growth conditions

Bacterial strains are listed in Table S1. *E. coli* NEB10beta (New England Biolabs) were used for assemblies and cloning procedures. *E. coli* BW29427 [*thrB1004 pro thi rpsL hsdS lacZ*ΔM15 RP4-1360 Δ(*araBAD*)567 Δ*dapA1341*::(*erm^+^ pir*^+^)] (K. Datsenko and B.L. Wanner, unpublished) was used for conjugation of plasmids into recipient bacteria. BW29427 requires supplementation with dl-α,ε-diaminopimelic acid (0.3 mM) for growth.

*F. novicida* MFN245 (25) was grown in tryptic soy broth supplemented with 0.1% (w/v) L-cysteine at 37°C on roller drum or on TS agar plates. *A. baumannii* ATCC 17978, *E. coli*, and *S. oneidensis* MR-1 were grown in LB or on LB agar plates. *V. alginolyticus* VIO5 was grown on VC media [0.5% (w/v) tryptone, 0.5% (w/v) yeast extract, 0.4% (w/v) K_2_HPO_4_, 3% (w/v) NaCl, 0.2% (w/v) glucose] (26). *A. baumannii* and *E. coli* were grown at 37°C, while *V. alginolyticus* and *S. oneidensis* were grown at 30°C. As needed, antibiotics were added to the growth medium at the following concentrations: kanamycin (50 µg/mL), chloramphenicol (20 or 2 µg/mL for *V. alginolyticus*), erythromycin (150 µg/mL), and carbenicillin (200 µg/mL).

### Cloning

Primers used in this study are listed in Table S2 and synthesized by Integrated DNA Technologies. DNA fragments were PCR-amplified with Platinum SuperFi II DNA Polymerase (Invitrogen) according to the manufacturer’s protocol. The PCR products were separated via agarose gel electrophoresis and purified with the Monarch DNA Gel Extraction Kit (NEB) according to the manufacturer’s protocol. Concentrations of gel-purified products were determined via Nanodrop (Thermo Scientific) prior to assembly reactions. Golden Gate cloning reactions were performed using NEBridge Ligase Master Mix with either *BsaI*-HFv2, *BbsI*-HF, or *PaqCI* as indicated. “Part” vectors were assembled with 0.05 pmols of each fragment within a single reaction. For final vectors, 0.05 pmols of each chromophore fragment were combined with 0.025 pmols of designated donor and destination plasmids. Assemblies were performed in a thermocycler under the following conditions: 37°C 1 min and 16°C 1 min for 30 cycles, followed by 65°C 5 min. Reactions were subsequently transformed into *E. coli* 10-beta cells (NEB) according to the manufacturer’s protocol, followed by plating on appropriate selective media.

### Plasmid construction

Construction of the “Part” and final plasmids is described in detail in the supplemental material. The final pKEK-Chrom plasmid set is listed in Table 2, and complete sequences are provided in supplementary material File S2.

**TABLE 2:** pKEK-Chrom plasmid series

Protein	Plasmid backbone	Marker	Plasmid designation	Addgene
eforCP/RFP	RSF1010-p15a	KanR	pKEK3223	234085
		CmR	pKEK3393	234077
	p15a-Fn Ori	KanR	pKEK3229	234101
		CmR	pKEK3193	234093
YukonOFP	RSF1010-p15a	KanR	pKEK3227	234089
		CmR	pKEK3395	234079
	p15a-Fn Ori	KanR	pKEK3233	234105
		CmR	pKEK3197	234095
aeCP597 (Blue) chromoprotein	RSF1010-p15a	KanR	pKEK3228	234090
		CmR	pKEK3396	234080
	p15a-Fn Ori	KanR	pKEK3234	234106
		CmR	pKEK3198	234096
tsPurple chromoprotein	RSF1010-p15a	KanR	pKEK3226	234088
		CmR	pKEK3394	234078
	p15a-Fn Ori	KanR	pKEK3232	234104
		CmR	pKEK3199	234097
DasherGFP	RSF1010-p15a	KanR	pKEK3225	234087
		CmR	pKEK3399	234083
	p15a-Fn Ori	KanR	pKEK3231	234103
		CmR	pKEK3194	234094
FuGFP	RSF1010-p15a	KanR	pKEK3224	234086
		CmR	pKEK3397	234081
	p15a-Fn Ori	KanR	pKEK3230	234102
		CmR	pKEK3200	234098
sfGFP	RSF1010-p15a	KanR	pKEK3237	234091
		CmR	pKEK3400	234084
	p15a-Fn Ori	KanR	pKEK3235	234107
		CmR	pKEK3220	234099
sfCherry2	RSF1010-p15a	KanR	pKEK3238	234092
		CmR	pKEK3398	234082
	p15a-Fn Ori	KanR	pKEK3236	234108
		CmR	pKEK3221	234100

### Conjugation

pKEK-Chrom plasmids were transformed into chemo-competent *E. coli* BW29427 (27). Successful transformants were grown overnight in LB containing 0.3 mM daptomycin (DAP) + appropriate antibiotics at 37 ℃. Recipient strains were grown overnight under appropriate conditions. Donor and recipient strains were pelleted separately at 12,000 rpm for 2 min, washed with phosphate-buffered saline (PBS) to remove residual media and antibiotics, and combined in a 1:1 ratio in 100 µL of PBS. Subsequently, 50 µL of donor:recipient mixtures was spotted on TSA plates supplemented with 0.3 mM DAP and incubated for 12–18 h under appropriate conditions for recipient strain. Mixtures were streaked for isolation on selective media in the absence of DAP and incubated under appropriate conditions to isolate single colonies.

### Promoter swap

A constitutive *Francisella*-specific promoter, p*groEL* (FTN_1539), was amplified from pKEK1140 (28) with primers GroEL Mob GG bsaI cgac F + Full groEL v2 R. After purification, 0.05 pmols of pGroEL PCR was combined with 75 ng of pKEK-Chrom-pACYC-p15A-KanR plasmids and assembled via the Golden Gate assembly with *BsaI*. Plasmids were transformed into *F. novicida* strain MFN245 (25) via electroporation (20) or conjugation, as described above with additional supplementation of 0.1% (w/v) L-cysteine and incubated at 37 ℃ for 24 or 48 h for conjugation or transformation, respectively.

### Fluorescence imaging

Petri plates were imaged on Singer Instruments PhenoBooth+. Plates were imaged under white, blue, and UV light. For green fluorescence, blue light (465–475 nm, peak ~470 nm) with 25 mm green emission filter was used for imaging. For red fluorescence, UV light (375–400 nm, peak ~390 nm) with a 25 mm red emission filter was used for imaging.

For fluorescence microscopy, *E. coli* harboring pKEK-Chrom-CmR-p15a-FnOri-oriT plasmids were grown overnight in LB media containing chloramphenicol (20 µg/mL) at 37°C. Cultures were diluted 1:100 in LB media plus chloramphenicol and grown to mid-log. Next, 1 mL of culture was pelleted and washed with 200 µL of sterile PBS. Then, 20 µL of the cell suspension was imaged on an agarose pad (29) with an Olympus BX43 fluorescent microscope equipped with a Hamamatsu ORCA-spark digital camera with Olympus U-FGNA 540–550 nm narrow bandpass filter and Olympus U-FBNA 470–495 nm narrow bandpass filter.

## RESULTS

### pKEK-Chrom vector palette plasmids replicate and express CCPs in diverse gram-negative bacteria

To ensure the versatility of our plasmid set across a broad range of host bacteria and applications, we selected a palette of eight CCPs (Table 1): eforCP, Free-use GFP (FuGFP), YukonOFP, DasherGFP, super-folder GFP (sfGFP), super-folder Cherry (sfCherry2), tinsel purple (tsPurple), and aeBlue (aeCP597). EforCP or chromo-red is a coral-derived chromoprotein originating from *Echinopara forskaliana* displaying spectral and phenotypic characteristics to red-fluorescent proteins (RFP) (3). FuGFP (iGEM BBa_K3814004) developed by Mark Somerville and Nick Coleman is an off-patent super-folder GFP variant that absorbs long-wave UV light and emits green, enabling a clearer separation between excitation and emission spectra and minimal overlap with RFPs (6). YukonOFP is a non-*Aequorea* orange fluorescent protein with a distinct emission spectrum from GFP proteins, which results in a visible orange color upon expression in bacterial species (8, 11). DasherGFP is a non-*Aequorea* monomeric fluorescent protein with excitation and emission wavelengths of 505 and 525 nm, respectively, and has shown to be functional in both prokaryote and eukaryote expression systems (5, 11, 30). CCP aeBlue (aeCP597) is a non-fluorescent chromoprotein derived from *Actinia equina* that has been codon-optimized for bacterial expression and reported to display temperature dependency for color development (4, 7, 31). TsPurple, a non-fluorescent chromoprotein, shares 91% of its protein coding sequence with aeBlue but develops into a distinct, rich purple color visible on agar plates without severely affecting fitness. This purple phenotype has led to its use in genetic tools for screening similar to *lacZ*-dependent blue-white screening (4, 32). EforCP, FuGFP, tsPurple, aeBlue, YukonOFP, and DasherGFP are all intellectual property-free, whereas sfGFP and sfCherry2 are patented chromophores engineered for enhanced fluorescence and stability in fusion and split fluorescent proteins (9, 10). This palette of eight CCPs will allow researchers to choose the most appropriate tool for their organisms and experimental setups.

**Fig 1:**
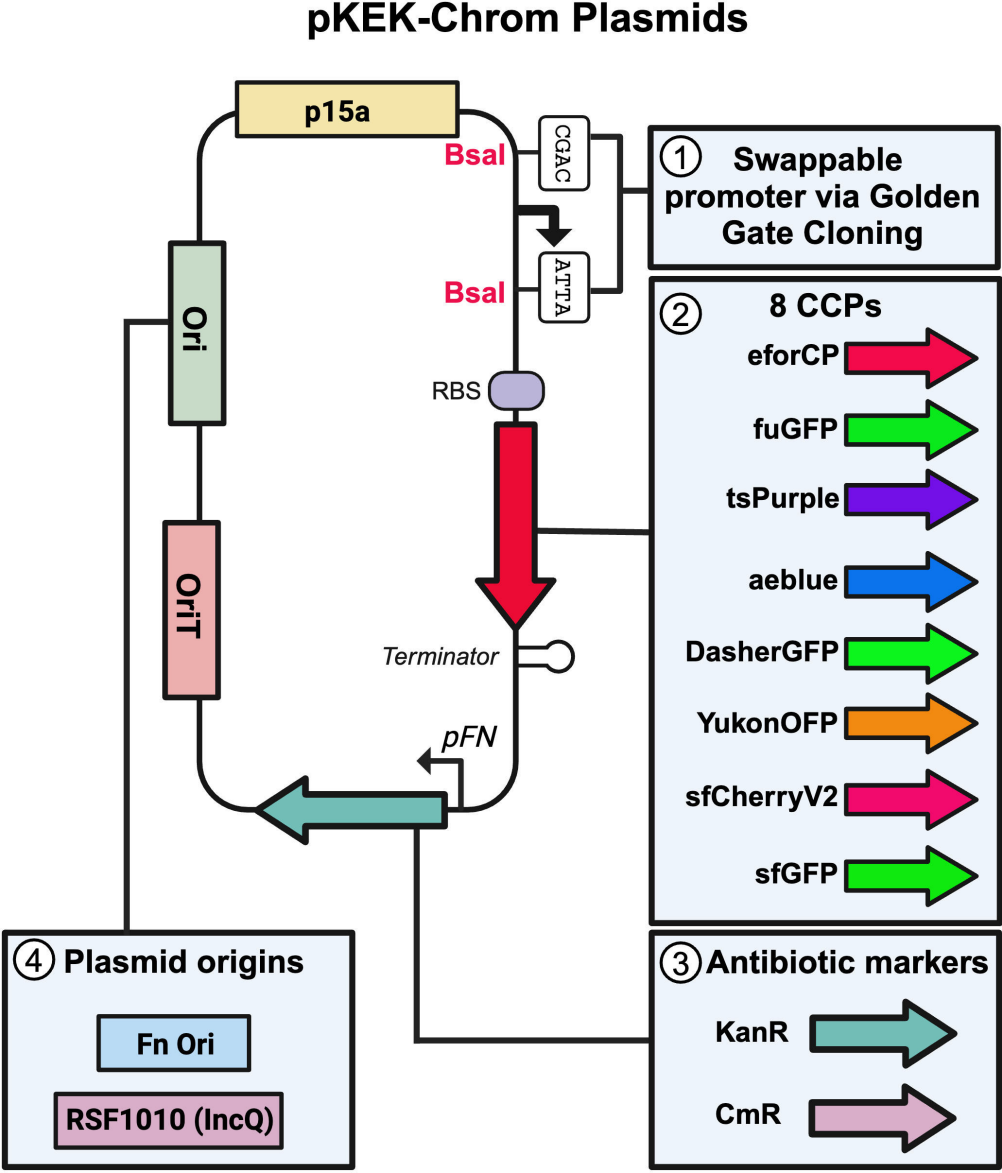
Schematic of the pKEK-Chrom plasmid series. This schematic demonstrates the critical components that enable CCP expression in diverse gram-negative bacteria, including (1) swappable promoter driving protein expression, (2) a variety of CCPs, (3) two different antibiotic resistance markers, and (4) two different options for broad host origins of replication. CCP = chromophore-containing protein, RBS = ribosome binding site, and pFN = pFN_1451 (18). Created in BioRender.

Using these CCPs, we constructed a plasmid set consisting of a total of 32 plasmids (Table 2), with four major attributes designed to broaden the applicability of our plasmids: (i) a promoter driving CCP expression that can be easily swapped via Golden Gate cloning; (ii) expression of eight different CCPs; (iii) two selectable antibiotic resistance markers (kanamycin and chloramphenicol resistance); and (iv) two different backbones with alternative origins of replication enabling replication in diverse hosts (Fig. 1).

The synthetic *pJ23100* (iGEM BBa_J23100) promoter, constructed by J.C. Anderson, has been optimized for constitutive, high-level expression in *E. coli* (33). We incorporated unique *BsaI* restriction sites flanking *pJ23100-BsaI*, which allows for this promoter to be easily swapped out for a different promoter by Golden Gate cloning. p*J23100*-BsaI was combined with a strong ribosome binding site to drive CCP expression in all pKEK-Chrom plasmids.

Since different bacteria may necessitate different antibiotic selective markers, we constructed vectors with two different markers conferring resistance to either kanamycin or chloramphenicol (Fig. 1). To ensure broad host-range applicability, we utilized two different backbones with the p15a origin of replication fused to either (i) an origin derived from the *Francisella* cryptic plasmid (FnOri) or (ii) the broad-host range origin, RSF1010 (34, 35). The p15a-FnOri was further fused to oriT (p15a-FnOri-oriT) to allow for mobilization via conjugation (36, 37). The RSF1010 ori contains an oriT element that enables mobilization to recipient strains, so oriT was not added to this backbone (p15a-RSF1010) (34).

Overall, the pKEK-Chrom plasmid set consists of 32 plasmids that enable expression of eight different CCPs in a wide range of host bacteria. The entire plasmid set is listed in Table 1 and deposited in Addgene (Table 2). We anticipate that the pKEK-Chrom plasmid series will provide a starting point to study CCP expression in non-model bacteria.

### Evaluation of CCP expression in gram-negative bacteria

**Fig 2:**
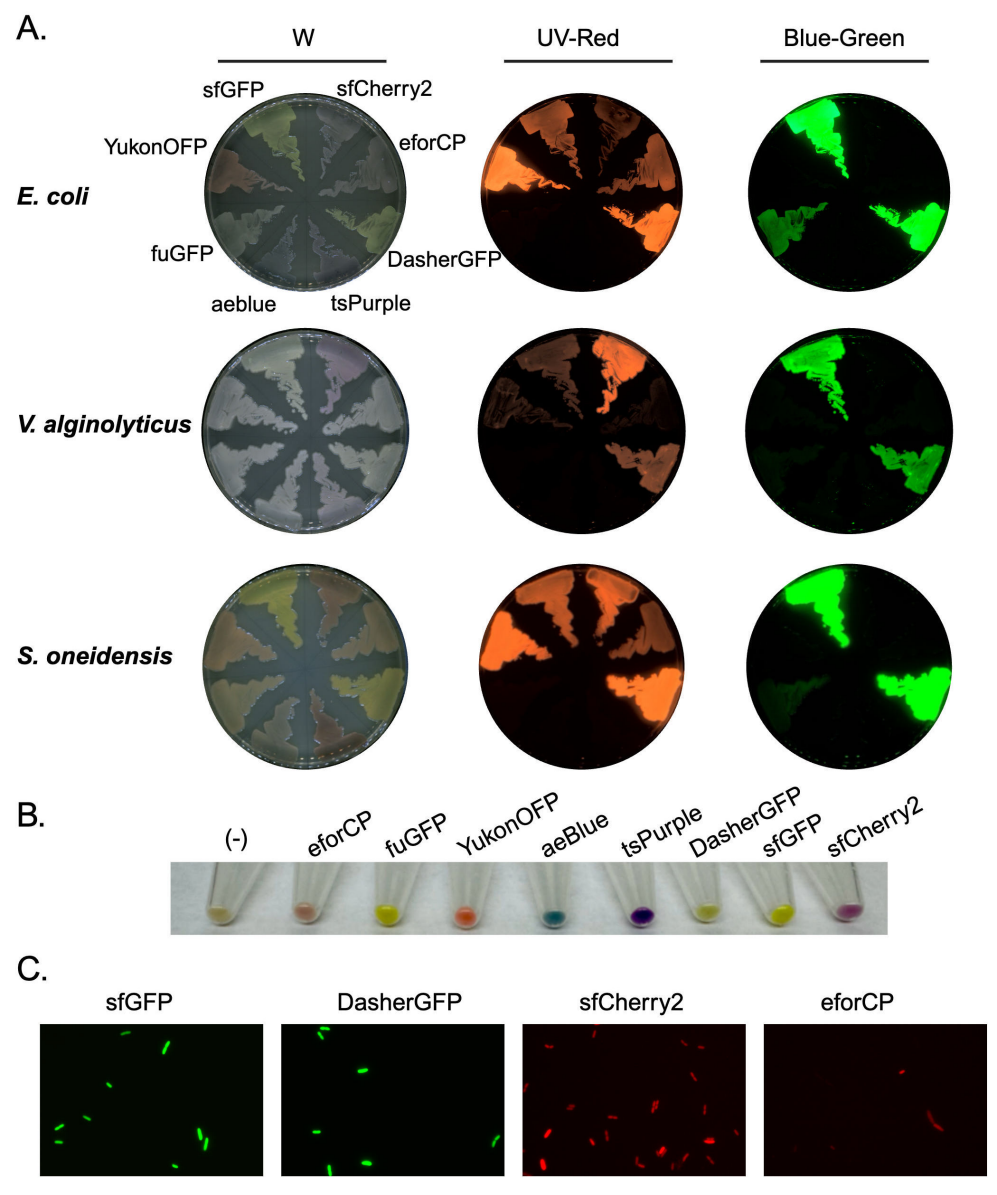
Expression of CCPs in diverse gram-negative bacteria. Expression of fluorescent proteins from the pKEK-Chrom-p15a-FnOri plasmid series in *E. coli*, *V. alginolyticus*, and *S. oneidensis* harboring expression vectors for eforCP (pKEK3193), DasherGFP (pKEK3194), tsPurple (pKEK3199), aeBlue (pKEK3198), YukonOFP (pKEK3197), sfGFP (pKEK3220), and sfCherry2 (pKEK3221). (A) Strains grown on agar plates and visualized under white (“W”), red (“UV-Red”), or green (“Blue-green”) filter. (B) Pellets of *Escherichia coli* strains from panel A grown in liquid media. (C) *E. coli* strains from panel A expressing sfGFP, DasherGFP, sfCherry2, and eforCP visualized via fluorescence microscopy. Created in BioRender.

*E. coli*, *V. alginolyticus*, and *S. oneidensis* can stably maintain plasmids containing the p15a origin of replication but have distinct growth requirements (e.g, temperature, halophilic media, etc.). We evaluated all eight of the pKEK-Chrom-p15a-FnOri-CmR plasmids for CCP expression in these strains (Fig. 2). Since the plasmids are identical, with the exception of the specific CCP, they can be compared to each other across species. As expected, all eight CCPs within our selected palette were expressed within *E. coli* strains harboring pKEK-Chrom plasmids. The *pJ23100-BsaI* promoter drove CCP expression to levels sufficient for visualization of both fluorescent and non-fluorescent chromoproteins within *E. coli* (Fig. 2A). In the red channel, fluorescence was detected for the RFP-like CCP eforCP, sfcherry2, and YukonOFP, as well as fluorescence given off by both sfGFP and DasherGFP. In the green channel, fluorescence was detected for sfGFP, DasherGFP, and FuGFP. Although the fluorescence of FuGFP was reduced compared to sfGFP and DasherGFP, FuGFP could not be detected in the red channel, unlike sfGFP and DasherGFP. eforCP, sfcherry2, and YukonOFP only fluoresced in the red channel.

In contrast to *E. coli*, *V. alginolyticus* is a halophilic bacterium that requires high salt-containing media and growth at 30°C. Although all eight pKEK-Chrom-p15a-FnOri-CmR plasmids were successfully introduced into *V. alginolyticus* by conjugation, observable CCP in *V. alginolyticus* displayed differences from what was seen in *E. coli*. Specifically, sfCherry2 fluoresced exceptionally well in *V. alginolyticus*, while YukonOFP, eforCP, and FuGFP were essentially not detectable (Fig. 2A). *S. oneidensis* also requires growth at 30°C and additionally displays a natural red hue. The FuGFP was barely detectable in *S. oneidensis*, indicating that this CCP may be limited to use in *E. coli* and closely related bacteria. In contrast to *V. alginolyticus*, eforCP and YukonOFP were detectable in *S. oneidensis*, and sfGFP and DasherGFP gave strong signal in both red and green channels. Thus, DasherGFP and sfGFP are good choices for green fluorescence in all three bacteria. YukonOFP and eforCP are good red fluorescence choices for *E. coli* and *S. oneidensis* but not *V. alginolyticus*, in which only sfCherry2 gave red fluorescence-specific signal.

To visualize the entire CCP palette offered by the pKEK-Chrom plasmid set (in addition to the fluorescent proteins), we concentrated *E. coli* cultures by centrifugation. Pelleted strains harboring pKEK-Chrom-p15a-FnOri-CmR expressing both fluorescent and non-fluorescent chromoproteins were clearly expressing the specific colors associated with the proteins (Fig. 2B). In the case of the chromoproteins aeBlue and tsPurple, the change in color could be readily observed (4).

sfGFP and sfCherry2 are patent protected due to their intentional engineering to provide improved characteristics for use in molecular research applications (10). In contrast, eforCP, aeblue, tsPurple, YukonOFP, fuGFP, and DasherGFP are intellectual property (IP)-free, which may broaden their potential use for bioindustrial purposes. Additionally, eforCP has been reported to have a unique spectral phenotype that is intermediate between non-fluorescent chromoproteins and typical red-fluorescent proteins (3). Thus, we sought to compare the IP and IP-free green fluorescent proteins while additionally comparing an optimized red fluorescent protein to a unique Chromo-Red fluorescent protein via fluorescence microscopy in *E. coli* (Fig. 2C). Both sfGFP and DasherGFP emitted similar levels of strong green fluorescence. In contrast, eforCP fluorescence was much weaker than sfCherry2 under our standard fluorescence microscopic conditions.

### Promoter swapping improves CCP expression in *F. novicida*

**Fig 3:**
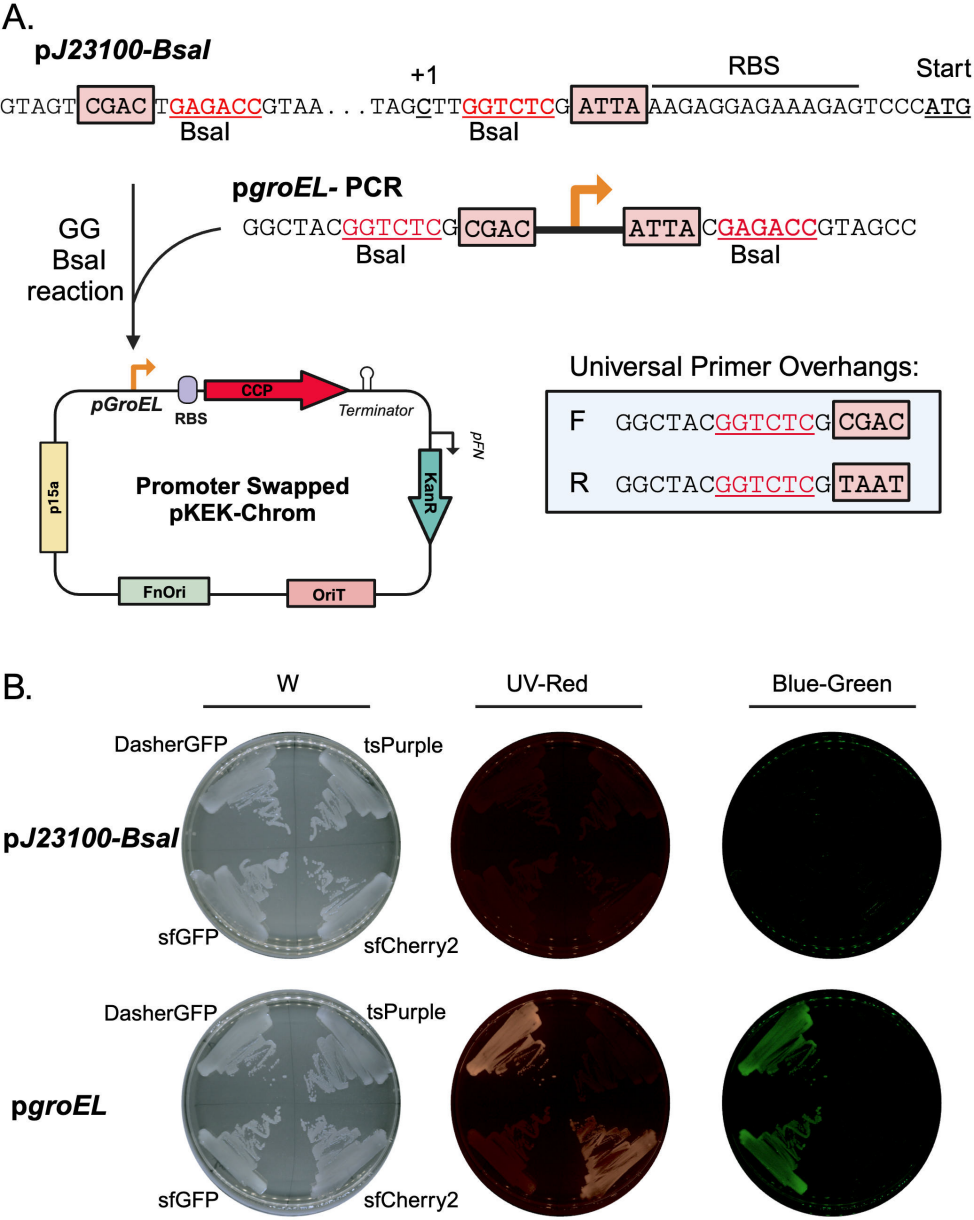
Promoter swap enables CCP expression in *Francisella*. The promoter element p*J23100*-BsaI in the pKEK-Chrom plasmid series enables promoter swapping in one-step Golden Gate reaction using circular pKEK-Chrom plasmids. (A) The schematic outlines how the p*J23100* promoter was swapped with the *Francisella* p*groEL* via the Golden Gate assembly using *BsaI*. The location of *BsaI* recognition sites within pKEK-Chrom plasmids are indicated in red; the resulting overhangs that guide ligation are boxed; the universal primer sequence to be used to amplify alternative promoters is indicated; and a schematic with the resulting promoter-swapped plasmid product is shown. (B) *F. novicida* strains harboring either p*J23100*- (top) or p*groEL* (bottom)-driven expression of sfCherry2 (pKEK3236 or pKEK3377), sfGFP (pKEK3235 or pKEK3372), DasherGFP (pKEK3231 or pKEK3310), and tsPurple (pKEK3232 or pKEK3311) grown on agar plates and visualized under white (“W”), red (“UV-Red”), or green (“Blue-green”) filter. Created in BioRender.

*Francisella* spp. are more distantly related to *E. coli*, *V. alginolyticus*, and *S. oneidensis* and do not transcribe most *E. coli* optimized promoters. The pKEK-Chrom plasmid series that contains the *Francisella* origin (FnOri) replicated in *F. novicida* but did not express detectable fluorescent proteins (Fig. 3B). This allowed us to demonstrate the usefulness of the swappable promoter element incorporated into these plasmids. The p*J23100* promoter element is flanked by unique type IIS restriction enzyme *BsaI* sites (GGTCTC). *BsaI* restriction digestion removes p*J23100* and leaves site-specific overhangs, 5′-CGAC-3′ and 5′-ATTA-3′ (Fig. 3A). Incorporating *BsaI* sites with complementary sequences into primers used to PCR amplify alternative promoter elements allows the fragments to be combined with any pKEK-Chrom plasmid in a one-step Golden Gate reaction to swap the promoter elements.

We validated promoter swapping in *F. novicida* (Fig. 3) by first selecting the *Francisella*-specific promoter, p*groEL*, which has been previously characterized for function in both *E. coli* and *F. novicida* (13, 14, 16, 17). The p*groEL* promoter element was amplified using primers containing the universal primer overhangs shown in Fig. 3A. The p*groEL* fragment was combined with pKEK-Chrom (p15a-FnOri) plasmids in individual Golden Gate reactions (*BsaI*) and subsequently evaluated for CCP expression in *F. novicida* (Fig. 3). The p*groEL-*swapped plasmids facilitated expression of CCPs in *F. novicida*. We selected p*groEL-*DasherGFP (pKEK3231), p*groEL*-sfGFP (pKEK3235), p*groEL*-tsPurple (pKEK3232), and p*groEL*-sfCherry2 (pKEK3236) for comparison (Fig. 3B). The *pJ23100* promoter was insufficient to drive the expression of any CCP in *Francisella* despite the stable maintenance of the plasmid (Fig. 3B). Plasmids isolated from *Francisella* strains harboring the p*J23100*-driven pKEK-Chrom plasmids expressed CCP upon re-transformation into *E. coli*, demonstrating that the lack of CCP visualization in *F. novicida* was likely due to host-specific transcriptional requirements (data not shown). In contrast, pGroEL enabled observable fluorescence of DasherGFP, sfGFP, and sfCherry2 and visualization of the chromoprotein, tsPurple. Expression of *pgroEL*-sfgfp and *pgroEL*-DasherGFP was detectable in both green and red channels. Expression of *pgroEL*-sfcherry2 in *F. novicida* was detectable strictly in the red channel.

We additionally assessed promoter swapping using a high-throughput pooled approach in which plasmids with eight different CCPs, pKEK3229 (eforCP), pKEK3230 (FuGFP), pKEK3231 (DasherGFP), pKEK3232 (tsPurple), pKEK3233 (YukonOFP), pKEK3234 (aeBlue), pKEK3235 (sfGFP), and pKEK3236 (sfCherry2), were combined with amplified p*groEL* in a single Golden Gate reaction. The reaction mixture was transformed directly into *F. novicida* strain MFN245 (25), and colonies with visible color changes or fluorescence after 48–72 h were selected and plasmids sequenced to confirm the presence of p*groEL*. Using this approach, five of the eight CCPs, sfGFP, sfCherry2, DasherGFP, tsPurple, and YukonOFP, were sufficiently expressed from p*groEL* in *F. novicida* to be identified from a single transformation. In contrast, the p*groEL*-driven expressions of aeBlue, FuGFP, and eforCP were not identified from the transformed pool presumably because these did not result in detectable color changes in *F. novicida*. Overall, this approach enabled the rapid identification of CCPs sufficiently expressed in *F. novicida* to obtain five of the eight CCP expression plasmids from a single Golden Gate reaction. Thus, these plasmids enable efficient promoter swapping to adapt them for use in different bacteria, and similar high-throughput pooled approaches may serve to rapidly identify appropriate promoters in other bacteria.

### The RSF1010 origin enables CCP expression in *A. baumannii*

The pKEK-Chrom plasmid series utilized in *E. coli*, *S. oneidensis*, *V. alginolyticus*, and *F. novicida* described above contain the p15a and FnOri origins of replication, along with oriT for conjugation. Multiple attempts were made to introduce these plasmids both by conjugation, as well as transformation, into *A. baumannii* to no avail. To broaden the utility of the pKEK-Chrom series even further, we constructed plasmids with the broad host range ori RSF1010 in conjunction with the p15a ori. RSF1010, originally isolated from *E. coli*, has been extensively investigated for use in plasmid shuttle vectors due to its ability to be mobilized and replicate in diverse bacteria (34). The RSF1010 origin encodes a native mobilizable (Mob) element and origin of transfer (oriT) shown to be efficiently mobilized by horizontal transfer to diverse bacteria (35, 38). The failure of the p15a-FnOri plasmids to replicate in *A. baumannii* allowed us to test the alternative RSF1010-p15a plasmids; *A. baumannii* has been previously shown to stably maintain RSF1010-based vectors (24, 39, 40).

**Fig 4:**
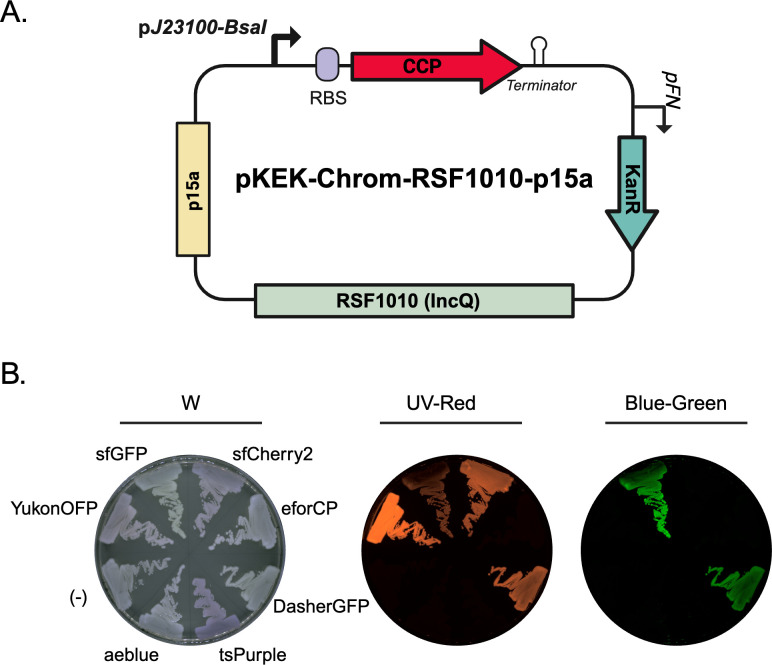
Alternate *ori* RSF1010 broadens host range and facilitates CCP expression in *A. baumannii*. (A) Schematic showing the plasmid structure of the pKEK-Chrom-RSF1010-p15a plasmid set evaluated in *A. baumannii*. (B) *A. baumannii* strains harboring plasmids expressing eforCP (pKEK3273), DasherGFP (pKEK3225), tsPurple (pKEK3226), aeblue (pKEK3228), Yukon (pKEK3227), sfGFP (pKEK3237), and sfCherry2 (pKEK3238), as well as *A. baumannii* with no fluorescent protein (“−”) were grown on agar plates and visualized under white (“W”), red (“UV-Red”), or green (“Blue-green”) filter. FP = fluorescent protein. Created in BioRender.

The pKEK-Chrom-RSF1010-p15a-KanR plasmids (Fig. 4A) were mobilized into *A. baumannii* via conjugation. Seven out of eight CCP-expressing plasmids were successfully conjugated into *A. baumannii* and evaluated for expression driven by the p*J23100-BsaI* promoter (Fig. 4B). Interestingly, the FuGFP-expressing plasmid was unable to be conjugated and stably maintained in *A. baumannii* after five independent attempts. YukonOFP and sfCherry2 fluoresced exclusively in the red channel, while sfGFP and DasherGFP fluoresced in the green channel, with overlap into the red channel. These results demonstrate that the p*J23100* promoter functions in *A. baumannii*, and that the inclusion of the RSF1010 origin broadens the potential host species of the pKEK-Chrom plasmid set.

## DISCUSSION

Since the discovery of GFP, a diverse range of CCPs comprising both fluorescent (e.g., GFP, RFP, and OFP) and non-fluorescent (e.g., aeBlue and tsPurple) chromophores has been identified and characterized and is now routinely utilized in microbiological research. In these applications, CCPs are typically expressed using plasmids, which require suitable promoters to drive CCP expression and origins of replication to ensure stable maintenance within the bacterial host. However, incorporating CCP-dependent assays in research on non-model bacterial species often presents challenges due to limitations in transformation methods, the need for organism-specific promoter activity, and suitable plasmid origins of replication. Addressing these issues can be time-consuming and resource-intensive.

In this study, we developed a versatile set of 32 plasmid vectors collectively referred to as the pKEK-Chrom plasmids, which can be readily modified to incorporate bacterial-specific elements, such as promoter sequences and plasmid origins of replication (Table 2; Fig. 1). The pKEK-Chrom plasmids express eight different CCPs, two different antibiotic resistance markers, and two different plasmid backbones. Using pKEK-Chrom plasmids, we evaluated CCP expression in different gram-negative bacteria, including *E. coli*, *V. alginolyticus*, *S. oneidensis*, *F. novicida*, and *A. baumannii* (Fig. 2 to 4).

The pKEK-Chrom plasmid series was designed to offer a range of CCPs and includes a Golden Gate-compatible promoter element, an oriT, two selectable markers (kanamycin or chloramphenicol resistance), and either the p15a-FnOri or RSF1010-p15a plasmid backbones (Fig. 1). We selected eight distinct CCPs—eforCP, DasherGFP, YukonOFP, tsPurple, aeBlue, FuGFP, sfGFP, and sfCherry2—providing a diverse palette of fluorescent and chromoproteins with various spectral properties, as detailed in Table 1. Expression of each CCP is driven by an *E. coli*-optimized constitutive promoter, p*J23100*, which can be easily swapped by a Golden Gate reaction to evaluate alternative organism-specific promoter elements. To overcome barriers to efficient transformation, the pKEK-Chrom plasmids include an oriT to facilitate mobilization from conjugative donor strains. To broaden the potential host range, two alternative origins of replication (p15a-FnOri or RSF1010-p15a) provide options for multiple bacterial species. We created versions conferring either kanamycin or chloramphenicol resistance in both plasmid backbones.

The pKEK-Chrom series with p15a-FnOri backbone was stably maintained in *E. coli*. All eight CCPs were expressed sufficiently in *E. coli* for visualization on agar plates and fluorescent microscopy (Fig. 2A throgh C). sfCherry2, eforCP, and YukonOFP were readily detectable only under red fluorescence filters. However, sfGFP and DasherGFP emitted fluorescence detectable in both red and green channels, while FuGFP displayed only green fluorescence with no significant crossover into the red channel. More stringent detection filters would likely eliminate this crossover; however, under our conditions, the specificity of FuGFP, as well as sfCherry2, eforCP, and YukonOFP, suggests these CCPs would be advantageous for experiments involving both red and green fluorescent proteins.

Because the pKEK-Chrom-p15a-FnOri plasmids also contain *oriT*, they were efficiently conjugated into *V. alginolyticus* and *S. oneidensis* from *E. coli* strains with chromosomally integrated RP4::Mu (e.g. S17-1, SM10, BW29427) (41). In *V. alginolyticus* and *S. oneidensis*, sfGFP and DasherGFP were visible in the green channel, while sfCherry2 and YukonOFP, along with sfGFP and DasherGFP, were detectable in the red channel. Interestingly, eforCP was only detectable in *S. oneidensis* and not *V. alginolyticus*, while FuGFP was undetectable in both. Although FuGFP was derived from the sfGFP amino acid sequence, its reduced fluorescence compared to sfGFP in *V. alginolyticus* and *S. oneidensis* demonstrates how species-specific metabolic differences may influence CCP detection. Additionally, *V. alginolyticus* and *S. oneidensis* with the chromoprotein tsPurple showed a visible purple coloration, but the chromoprotein aeBlue exhibited only a slight blue tint potentially due to the previously described temperature dependency of the chromoprotein (31).

To further explore the versatility of the pKEK-Chrom plasmids, we investigated promoter swapping as a method to customize CCP expression. The incorporation of flanking *BsaI* restriction sites into the p*J23100* promoter enables its replacement with alternative promoters using Golden Gate cloning reactions with PCR fragments containing universal overhangs (Fig. 3A). The pKEK-Chrom p15a-FnOri plasmids containing the p*J23100* promoter were stably maintained in *F. novicida* but failed to express detectable CCPs. *Francisella* species often require *Francisella*-specific promoter elements due to their unique transcriptional machinery, so we swapped the p*J23100* promoter with the *Francisella*-specific promoter p*groEL* through Golden Gate cloning (Fig. 3). The introduction of the *Francisella* promoter allowed the detection of sfGFP, sfCherry2, tsPurple, and DasherGFP in *F. novicida* (Fig. 3B). aeBlue and FuGFP expressed from p*groEL* were undetectable in *F. novicida*, while YukonOFP and eforCP required an extended incubation period (>48 h) (data not shown). These findings demonstrate how easily promoter swapping in the pKEK-Chrom plasmids can customize species-specific expression requirements.

To demonstrate how to expand the host range of our plasmid set, we replaced the FnOri-*oriT* segment with the broad host range-origin RSF1010 (RSF1010-p15a series) (Fig. S1). The RSF1010 origin is mobilizable through conjugation, so the *oriT* element was removed in these plasmids. The RSF1010-p15a series of plasmids was generated by adding PCR fragments encoding all eight CCP genes to the other “part” vectors in a single Golden Gate assembly reaction, as detailed in the supplemental information (Fig. S2). From this single reaction, we were able to recover all eight individual CCP expression pKEK-Chrom-RSF1010-p15a-CmR plasmids, further highlighting the quick adaptability of these plasmids to specific host bacteria.

The pKEK-Chrom plasmids with RSF1010- were mobilized into and stably maintained in *A. baumannii* (Fig. 4). YukonOFP and sfCherry2 could be visualized in the red channel, while sfGFP and DasherGFP could be visualized in both green and red channels. Similar to *V. alginolyticus*, eforCP could not be detected in *A. baumannii*, and the chromoprotein, aeBlue, did not produce a noticeable color change (Fig. 4B). Interestingly, we were unable to introduce plasmids expressing FuGFP into *A. baumannii* despite multiple attempts. The inability to introduce FuGFP into *A. baumannii* suggests that this fluorescent protein may require further optimization for use in other non-model bacteria .

In summary, the pKEK-Chrom plasmid series is designed to enable the expression of CCPs across a wide range of gram-negative bacteria. The various differences in CCP activity we observed using the pKEK-Chrom plasmid series in each of the bacterial strains are summarized in Table S4. The use of eight different CCPs, two different origins of replication, two antibiotic selectable markers, and a modified Golden Gate-compatible promoter element provides the adaptability to ensure a broad potential host range for these plasmids while also allowing for easy modification to incorporate host-specific elements crucial for efficient CCP expression.

## Data Availability

All 32 pKEK-Chrom plasmids are available through Addgene (https://www.addgene.org/browse/article/28252870/) or upon request.

## SUPPLEMENTAL MATERIAL

File S1 aem.00045-25-s0001.docx Supplemental methods, Fig. S1 and S2, and Tables S1 to S4.

File S2 aem.00045-25-s0002.xlsx Sequences of all 32 plasmids within the pKEK-Chrom plasmid series.

ASM does not own the copyrights to Supplemental Material that may be linked to, or accessed through, an article. The authors have granted ASM a non-exclusive, world-wide license to publish the Supplemental Material files. Please contact the corresponding author directly for reuse.
