# Supplementary material for: Mobilizable shuttle vectors with fluorescent markers functional across different species of bacteria
Source: Appl Environ Microbiol. 2025 May 12;91(6):e00045-25. doi: 10.1128/aem.00045-25 (PMC12175534; doi:10.1128/aem.00045-25)
Supplement: File S1 — Supplemental methods, Fig. S1 and S2, and Tables S1 to S4. [file aem.00045-25-s0001.docx]

**Supplemental Information**

**Plasmid Construction:**

**“Part” vectors:** To facilitate assembly of CCP expression vectors, we first constructed several component, or “part” vectors for subsequent use in Golden Gate Cloning reactions. First, donor plasmids carrying antibiotic cassettes driven by the constitutive FTN_1451 promoter (*pFn*) were constructed by PCR-amplification of either KanR or CmR cassettes pKEK898 or pKEK923 (1), respectively, with primers Fn ab uni bbsI GG paqCI BsaI F and FnABuni GG bbsI gctt R (primers 1 and 2 in Fig S1). These primers incorporate flanking *PaqC*I sites for downstream assemblies. Primers pUC118 MCS bsbI GG F + OriT no paqCI bbsI mob gg R (primers 3 and 4 in Fig. S1), and oriT no paqCI mob bbsI gg F + pUC188 MCS bbsI gg R (primers 5 and 6 in Fig. S1) were used to PCR-amplify an R6K-containing plasmid backbone from pKEK2200 (R6K-CmR) or pKEK2201 (R6K-KanR) (2) and remove an internal *PaqC*I site within oriT. These fragments were subsequently assembled with either KanR or CmR via Golden Gate assembly with *BbsI* to create pKEK3061 and pKEK3062, respectively (“Antibiotic Donor Vectors” Fig. S1).

Two destination “part” vectors containing either p15a-FnOri-OriT (pKEK3064) or RSF1010-p15a (pKEK3063) were constructed in the following manner. Primers oriT mob gg bsaI no paqCI F + OriT no paqCI bsaI mob gg R (primers 7 and 8 in Fig. S1B) and oriT no paqCI mob gg F + pacyc BB bsaI gg R (primers 9 and 10 in Fig. S1B) were used to amplify the p15a-Fnori-oriT backbone from pKEK2960- a *Francisella* shuttle vector derived from pKK202 (3), and further modified to incorporate a mobilizable element (oriT) and Kanamycin resistance- and remove an internal *PaqC*I site within oriT. For RSF1010-p15a, primers RSF1010 bsaI gg BB FWD + RSF1010 bsaI UNI REV (primers 11 and 12 in Fig. S1B) were used to amplify the RSF1010 origin from pJB-CAT (4, 5), which includes the native RSF1010 mobilizable element, and pACYC GG RSF1010 F + pACYC BB bsaI gg R (primers 13 and 14 in Fig. S1B) were used to amplify p15a from pKEK2960 (described above). To facilitate selection and incorporate internal *PaqCI* sites for downstream assemblies, an antibiotic cassette conferring resistance to Erythromycin (ErmR) was amplified with Fn ab uni bsaI tagc BB F + Fn ab uni bsaI catc BB R from pKEK887 (primers 15 and 16 in Fig. S1B) (1). All backbone fragments were assembled via Golden Gate Assembly with *BsaI*, resulting in pKEK3063 and pKEK3064 (Fig. S1B-C).

Lastly, a modified p*J23100* (iGEM BBa_J23100) promoter (6) containing flanking *PaqCI* and *BsaI* sites for assembly and promoter swapping, respectively, was designed and synthesized by TWIST Biosciences (pKEK3212) (“pJ23100-BsaI Donor Vector” in Fig. S1C). The final part vectors and corresponding *PaqCI* overhangs are shown in Figure S1C.

The CCPs were originally cloned in the manner described below using information from the iGEM Parts Registry. CCPs eforCP (iGEM BBa_K592012), FuGFP (iGEM BBa_K3814004), YukonOFP (iGEM BBa_K1429003), DasherGFP (ATUM Protein PaintBox, Newark, CA, USA) (7), tsPurple (iGEM BBa_K1033905), and aeBlue (iGEM BBa_K864401) were maintained in pIDMv5K, a Kanamycin resistant derivative of pIDMv5 (NCBI:txid2968833). A modular traditional cloning scheme was used to construct the pIDMv5 vector. First, an upstream promoter element consisting of the *E. coli* optimized promoter elements, p*J23100* (iGEM BBa_J23100) and RBS (iGEM BBa_B0030), pJ23100-B0030, were synthesized by Twist Biosciences with flanking *EcoRI* and *NcoI* restriction sites and subsequently cloned into the plasmid backbone. Second, a rho-independent hairpin transcriptional terminator (iGEM BBa_B1006) containing flanking *SacI* and *KpnI* restriction sites was synthesized by TWIST biosciences and cloned into the pIDMv5K-pJ23100 via *SacI* and *KpnI*. The resultant vector, pIDMv5K-pJ23100-B1006, contains unique *NcoI* and *SacI* sites that enable downstream in-frame cloning of CCP genes. All 6 CCP genes were synthesized by TWIST biosciences based on available sequences with flanking *NcoI* and *SacI* restriction sites and subsequently cloned into the pIDMv5-pJ23100-B1006, resulting in 6 final pIDMv5-pJ23100-Chrom-B1006 plasmids (Table S3). All 6 plasmids are available at <https://atinygreencell.com/products/rainbow-chromoprotein-expression-plasmid-set>.

To enable the use of universal primers to amplify all 8 CCPs, PCR template vectors for sfGFP and sfCherry2 were created to reflect the same transcriptional organization as pIDMv5-p*J23100:* p*J23100-*Chrom-Term (iGEM BBa_B1006). First, aeBlue was amplified from pIDMv5-p*J23100*-aeBluewith primers pIDM chrom uni paqCI F + Chrom uni paqCI R and combined in PaqCI Golden Gate Reaction with part vectors pKEK3061 (KanR) and pKEK3064 (p15a-FnOri-OriT), generating pKEK3150 (pJ23100-aeBlue-B1006Term-KanR-OriT-FnOri-p15a). Next, primers pIDM sfgfp F+ pIDM sfgfp R, annealing to p*J23100* and transcriptional terminator, respectively, were used to amplify the pKEK3150 backbone. Both, sfGFP, amplified from pKEK2960 with primers sfgfp pIDM gg F + sfgfp pIDM gg R, and sfCherry2, amplified from linear DNA fragment synthesized by TWIST biosciences® with primers sfcherry pIDM F + sfcherry pIDM R, were subsequently cloned into the pKEK3150 backbone via Golden Gate assembly with *BsaI*, creating pKEK3210 (p*J23100*-sfGFP-Term) and pKEK3213 (p*J23100*-sfCherry2-Term).

**pKEK-Chrom plasmid assembly:**

For construction of final pKEK-Chrom plasmids, each chromophore was amplified with RBS uni gg paqCI FWD + Chrom uni paqCI R (primers 17 and 18 in Fig. S1D) and combined within a single Golden Gate reaction with 1. either pKEK3063 (RSF1010-p15a) or pKEK3064 (p15a-FnOri-OriT), 2. pKEK3061 (KanR) or pKEK3062 (CmR), 3. pKEK3212 (pJ23100-BsaI) and subsequently assembled with *PaqCI* (Fig. S1E). The antibiotic “part” donor plasmids have the R6K *ori* and cannot replicate in *E. coli* strains lacking *pir*, while the promoter donor plasmid is AmpR; thus, there was no background of “part” plasmids upon transformation of *E. coli* and selection with either KanR or CmR, and a single Golden Gate reaction with all eight CCP fragments typically yielded the correct eight CCP expression plasmids. Whole Plasmid Sequencing of part vectors was performed by Plasmidsaurus using Oxford Nanopore Technology with custom analysis and annotation.


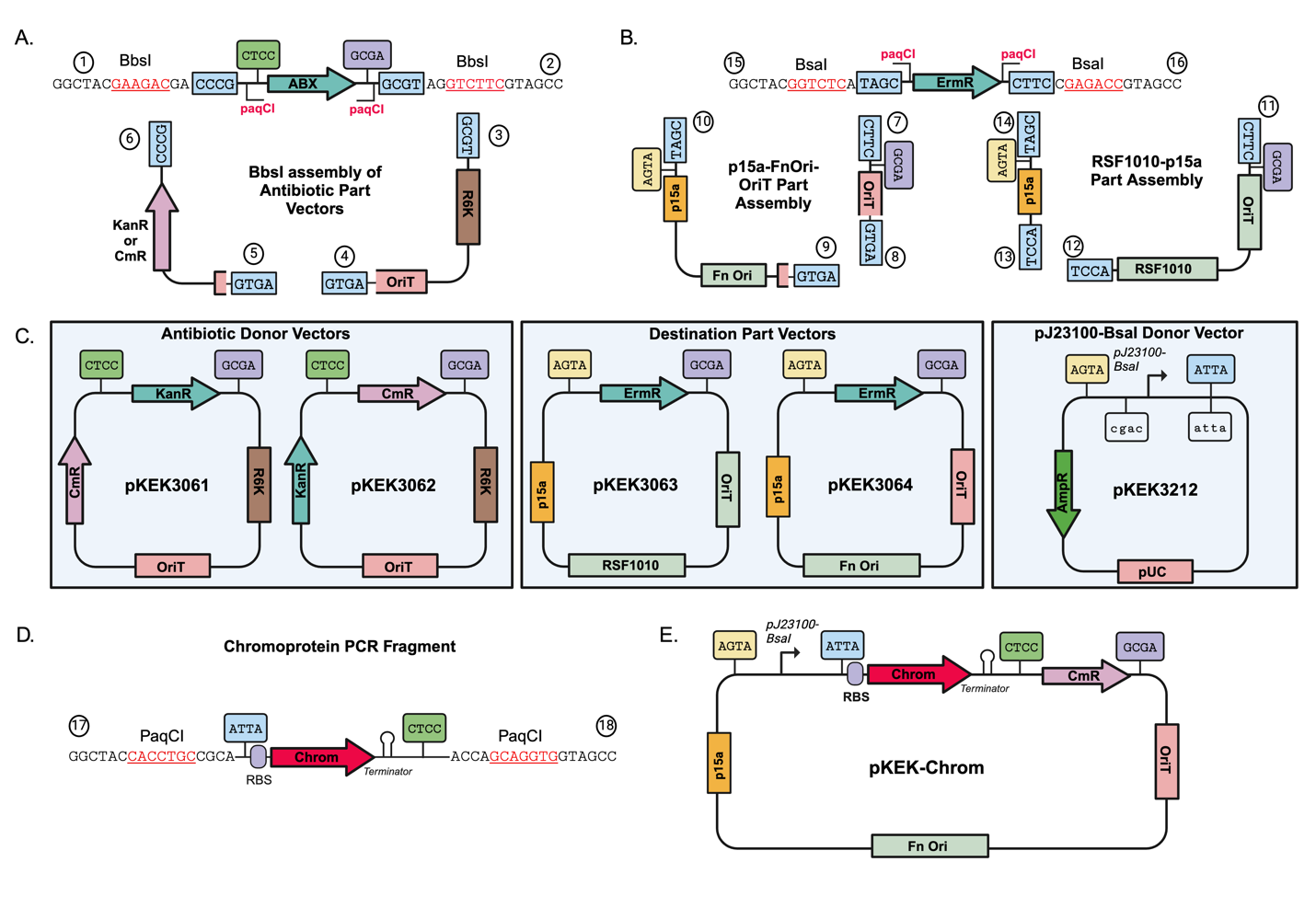


**Figure S1. Construction of pKEK-Chrom Component Vectors and Cloning Scheme.** (A) Construction of antibiotic (KanR or CmR) part vectors, (B) Construction of p15a-FnOri-OriT and RSF1010-p15a backbone part vectors, (C) Schematic of all part vectors and resulting overhangs after PaqCI digestion for assembly of various pKEK-Chrom plasmids, (D) Example of chromoprotein amplification with universal primers, and (E) Example of final pKEK-Chrom plasmid resulting from Golden Gate Assembly with PaqCI and parts: pKEK3062, pKEK3064, pKEK3212, and CCP PCR product. Primers used to amplify each of the part fragments are indicated by corresponding number. Overhangs resulting from either BbsI (A) or BsaI (B) are indicated in light blue boxes, and location, orientation, and resulting overhangs of PaqCI sites that mediate final pKEK-Chrom assembly are shown.


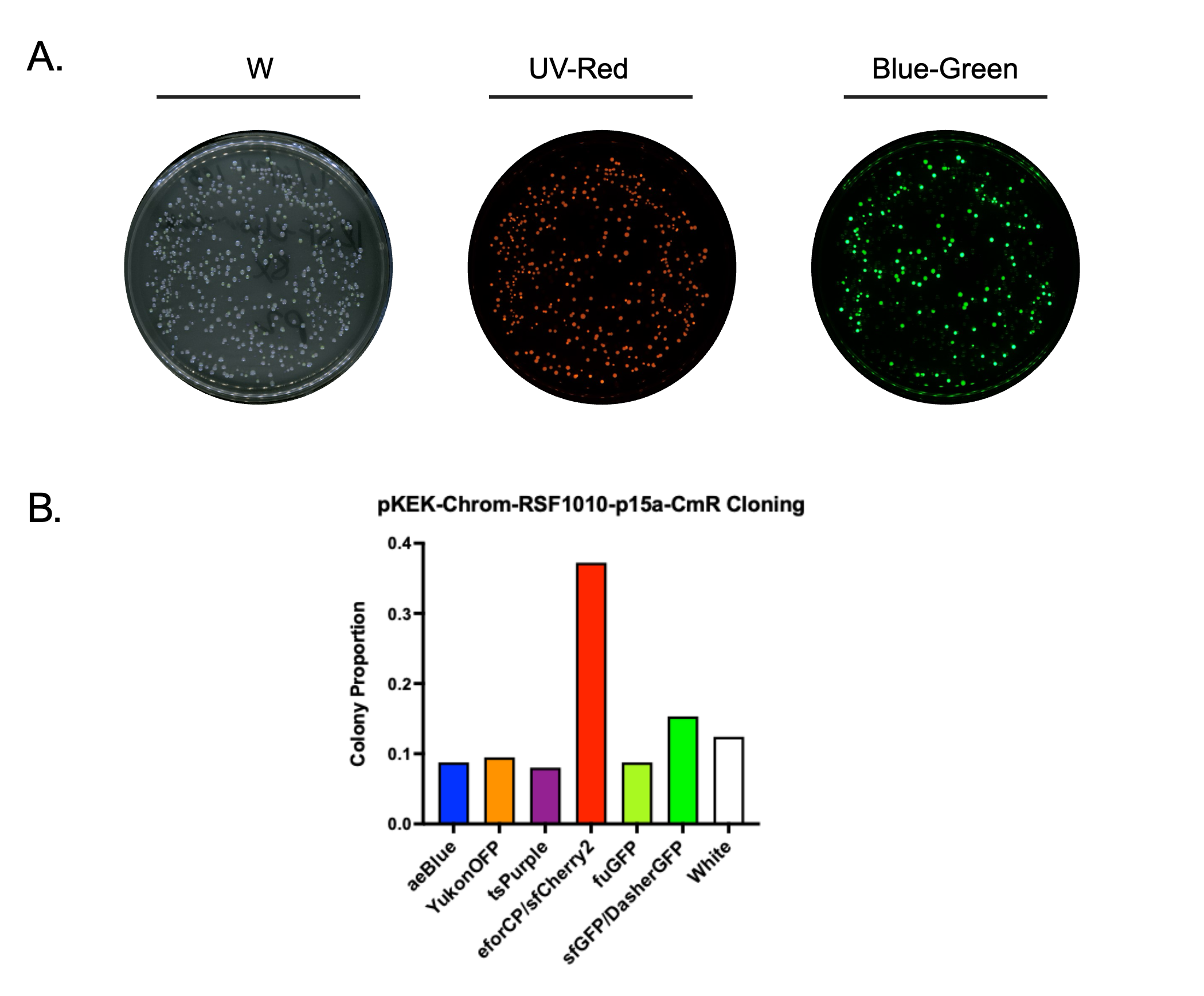


**Figure S2: Single Golden Gate Reaction Assembly Efficiency of 8 CCPs into pKEK-Chrom-RSF1010-p15a**. **A.** Visualization of CCP expression in *E. coli* NEB10beta transformants after 48-hour incubation. **B.** Representative proportions of clones visibly expressing each CCP within a single quadrant. EforCP/sfcherry2 and sfGFP/DasherGFP could not be distinguished based on fluorescence characteristics and are thus combined.

**Table S1. Bacterial strains used in this study**

| **Strain** | **Genotype** | **Source** |
| --- | --- | --- |
| *E. coli* NEB10beta | *Δ(ara-leu) 7697 araD139  fhuA ΔlacX74 galK16 galE15 e14-  ϕ80dlacZΔM15  recA1 relA1 endA1 nupG  rpsL (Str^R^) rph spoT1 Δ(mrr-hsdRMS-mcrBC)* | New England Biolabs |
| *E. coli* BW29427 | *thrB1004 pro thi hsdS lacZ*-ΔM15 RP4-1360 Δ(*araBAD*)*567* Δ-*dapA1341*::[erm pir(wt)] | K. Datsenko and B. L. Wanner |
| *Vibrio alginolyticus VIO5* | VIK4 laf (Rif^R^ Laf^-^) | (8) |
| *Shewanella oneidensis* MR-1 | Wild-type | Jeffrey Gralnick, University of Minnesota |
| *Francisella novicida* MFN245 | *hsdRI; hsdRII; res; drg* | (9) |
| *Acinetobacter baumannii* (ATCC17978) | Wild-type | **American Type Culture Collection** |

**Table S2. Primers used in this study**

| Name | Sequence (5’-3’) |
| --- | --- |
| Fn ab uni bbsI GG paqCI BsaI F | GGCTACGAAGACGA**CCCG**ACCACCTGCTGGTCTCCCCAACGGCGTAGAGGATCGAGATCT |
| FnABuni GG bbsI gctt R | GGCTACGAAGACC**TACG**CTACCACCTGCGGTCTCGCGCTTTGTTAGCAGCCGGATCC |
| pUC118 MCS bbsI GG F | GGCTACGAAGACC**TGCG**TAGCCTGCAGGCATGCAAGCTT |
| pUC188 MCS bbsI gg R | GGCTACGAAGACT**ACGG**GTACCGAGCTCGAATTC |
| oriT mob gg bsaI no paqCI F | GGCTACGGTCTCG**CTTC**GCGAGCAGCCGACCAGGCTTTCC |
| OriT no paqCI bsaI mob gg R | GGCTACGGTCTCA**TCAC**CTCCTGGTTAGCTTCCGCGCG |
| oriT no paqCI mob gg F | GGCTACGGTCTCA**GTGA**TAGGTCTTGTCGGCC |
| Fn ab uni bsaI tagc BB F | GGCTACGGTCTCA**TAGC**GCAGGTGCGGCGTAGAGGATCGAGATCT |
| Fn ab uni bsaI catc BB R | GGCTACGGTCTCG**GAAG**GCAGGTGCTTTGTTAGCAGCCGGATCC |
| RSF1010 bsaI UNI REV | GGCTACGGTCTCT**TGGA**GGTGAAGGGCAATCAGCTG |
| RSF1010 bsaI gg BB FWD | GGCTACGGTCTCC**CTTC**GCGAGCCTGTCAGACCAAGTTTACTCATATATACTTTAG |
| pACYC GG RSF1010 F | GGCTACGGTCTCA**TCCA**AGCTGTCCCTCCTGTTCAG |
| pacyc BB bsaI gg R | GGCTACGGTCTCG**GCTA**TACTCTAGAAATATTTTATCTGATTAATAAGATGATCTTCTTG |
| OriT no paqCI bbsI mob gg R | GGCTACGAAGACCT**TCAC**CTCCTGGTTAGCTTCCGCGCG |
| oriT no paqCI mob bbsI gg F | GGCTACGAAGACCT**GTGA**TAGGTCTTGTCGGCC |
| RBS uni gg paqCI FWD | GGCTACCACCTGCCGCA**ATTA**AAGAGGAGAAAGAGTCCCATG |
| Chrom uni paqCI R | GGCTACCACCTGCTGGT**GGAG**CAGGAAACAGCTATGACGGTACC |
| sfcherry pIDM F | GGCTACGGTCTCG**ATGG**AAGAAGATAATATGGCAATTATCAAGG |
| sfcherry pIDM R | GGCTACGGTCTCG**CTTA**TCATGTAGAGTGACGTGCCTC |
| sfgfp pIDM gg F | GGCTACGGTCTCG**ATGG**GCAGCAAAGGCGAAGAACTGTTTAC |
| sfgfp pIDM gg R | GGCTACGGTCTCG**CTTA**TCACTTGTCGTCGTCGTCC |
| pIDM sfgfp F | GGCTACGGTCTCG**TAAG**AGCTCAAAAAAAAACCCCGC |
| pIDM sfgfp R | GGCTACGGTCTCG**CCAT**GGGACTCTTTCTCCTCTTTAATC |
| GroEL Mob GG bsaI cgac F | GGCTACGGTCTCG**CGAC**GGCTGCTAACAAAGCCCG |
| Full groEL v2 R | GGCTACGGTCTCG**TAAT**GTCGAGAATGGACGAATGTTCATAAC |
| pIDM chrom uni paqCI F | GGCTACCACCTGCCGCA**AGTA**GTAAAACGACGGCCAGTGAATTC |
| Type IIS restriction site is underlined and resulting 4 nucleotide overhang is indicated in bold. | |

**Table S3: Plasmids used in this study**

| **Plasmid** | **Origin** | **Chromogen** | **Phenotype** | **Source** |
| --- | --- | --- | --- | --- |
| pKEK3061 | Kanamycin donor | N/A | KanR, CmR | This study |
| pKEK3062 | Chloramphenicol donor | N/A | CmR, KanR | This study |
| pKEK3063 | RSF1010 (IncQ)-p15a destination backbone | N/A | ErmR | This study |
| pKEK3064 | p15A-Fn Ori destination backbone | N/A | ErmR | This study |
| pKEK3212 | pJ23100-BsaI Donor | N/A | AmpR | This study |
| pIDMv5K-pJ23100-eforCP | PCR template | eforCP | KanR | (10) |
| pIDMv5K-pJ23100-tsPurple | PCR template | Tspurple | KanR | (10) |
| pIDMv5K-pJ23100-aeBlue | PCR template | aeBlue | KanR | (10) |
| pIDMv5K-pJ23100-DasherGFP | PCR template | DasherGFP | KanR | (10) |
| pIDMv5K-pJ23100-fuGFP | PCR template | FuGFP | KanR | (10) |
| pIDMv5K-pJ23100-YukonOFP | PCR template | YukonOFP | KanR | (10) |
| pKEK898 | p*Fn*-KanR Template | N/A | KanR, AmpR | (1) |
| pKEK923 | p*Fn*-CmR Template | N/A | CmR, AmpR | (1) |
| pKEK887 | p*Fn*-ErmR Template | N/A | ErmR, AmpR | (1) |
| pKEK2200 | R6K-CmR Template | N/A | CmR | (2) |
| pKEK2201 | R6K-KanR Template | N/A | KanR | (2) |
| pKEK2960 | p15a-Fnori-oriT- Template | N/A | KanR | This study |
| pJB-CAT | RSF1010 Template | N/A | CmR | (4, 5) |
| pKEK3150 | pIDMv5 backbone template | aeBlue | KanR | This study |
| pKEK1140 | pGroEL template | N/A | TetR | (11) |
| pKEK3210 | sfGFP template | sfGFP | KanR | This Study |
| pKEK3213 | sfCherry template | sfCherry2 | KanR | This Study |
| pKEK3393 | RSF1010-p15a | eforCP | CmR | This study |
| pKEK3394 | RSF1010-p15a | tspurple | CmR | This study |
| pKEK3395 | RSF1010-p15a | YukonOFP | CmR | This study |
| pKEK3396 | RSF1010-p15a | aeBlue | CmR | This study |
| pKEK3397 | RSF1010-p15a | FuGFP | CmR | This study |
| pKEK3398 | RSF1010-p15a | sfCherry2 | CmR | This study |
| pKEK3399 | RSF1010-p15a | DasherGFP | CmR | This study |
| pKEK3400 | RSF1010-p15a | sfGFP | CmR | This study |
| pKEK3223 | RSF1010-p15a | eforCP | KanR | This study |
| pKEK3224 | RSF1010-p15a | FuGFP | KanR | This study |
| pKEK3225 | RSF1010-p15a | DasherGFP | KanR | This study |
| pKEK3226 | RSF1010-p15a | tsPurple | KanR | This study |
| pKEK3227 | RSF1010-p15a | YukonOFP | KanR | This study |
| pKEK3228 | RSF1010-p15a | aeBlue | KanR | This study |
| pKEK3237 | RSF1010-p15a | sfGFP | KanR | This study |
| pKEK3238 | RSF1010-p15a | sfCherry2 | KanR | This study |
| pKEK3193 | Fn Ori-p15a | eforCP | CmR | This study |
| pKEK3194 | Fn Ori-p15a | DasherGFP | CmR | This study |
| pKEK3197 | Fn Ori-p15a | YukonOFP | CmR | This study |
| pKEK3198 | Fn Ori-p15a | aeBlue | CmR | This study |
| pKEK3199 | Fn Ori-p15a | tsPurple | CmR | This study |
| pKEK3200 | Fn Ori-p15a | FuGFP | CmR | This study |
| pKEK3220 | Fn Ori-p15a | sfGFP | CmR | This study |
| pKEK3221 | Fn Ori-p15a | sfCherry2 | CmR | This study |
| pKEK3229 | Fn Ori-p15a | eforCP | KanR | This study |
| pKEK3230 | Fn Ori-p15a | FuGFP | KanR | This study |
| pKEK3231 | Fn Ori-p15a | DasherGFP | KanR | This study |
| pKEK3232 | Fn Ori-p15a | tsPurple | KanR | This study |
| pKEK3233 | Fn Ori-p15a | YukonOFP | KanR | This study |
| pKEK3234 | Fn Ori-p15a | aeBlue | KanR | This study |
| pKEK3235 | Fn Ori-p15a | sfGFP | KanR | This study |
| pKEK3236 | Fn Ori-p15a | sfCherry2 | KanR | This study |
| pKEK3372 | Fn Ori-p15a | pGroEL-sfGFP (3235) | KanR | This study |
| pKEK3377 | Fn Ori-p15a | pGroEL-sfCherry2 (3236) | KanR | This study |
| pKEK3310 | Fn Ori-p15a | pGroEL-DasherGFP (3231) | KanR | This study |
| pKEK3311 | Fn Ori-p15a | pGroEL-tsPurple (3232) | KanR | This study |

**Table S4. Summary of CCP activity in diverse Gram-negative bacteria**

| Strain | Origin | Promoter | eforCP | Yukon  OFP | aeBlue | tsPurple | DasherGFP | FuGFP | sfGFP | Sf  Cherry2 |
| --- | --- | --- | --- | --- | --- | --- | --- | --- | --- | --- |
| *V. alginolyticus* | p15a-Fn Ori | p*J23100*-BsaI | - | - | + | + | + | - | + | + |
| *S. oneidensis* | p15a-Fn Ori | p*J23100*-BsaI | + | + | + | + | + | - | + | + |
| *F. novicida* | p15a-Fn Ori | p*GroEL* | - | + | - | + | + | - | + | + |
| *A. baumannii* | RSF1010-p15a | p*J23100*-BsaI | - | + | - | + | + | nd | + | + |

nd: not determined

**Supplemental References**

1. Liu J, Zogaj X, Barker JR, Klose KE. 2007. Construction of targeted insertion mutations in Francisella tularensis subsp. novicida. Biotechniques 43:487-90, 492.

2. Mejia-Santana A, Lloyd CJ, Klose KE. 2021. New cloning vectors to facilitate quick allelic exchange in gram-negative bacteria. Biotechniques 70:116-119.

3. Norqvist A, Kuoppa K, Sandstrom G. 1996. Construction of a shuttle vector for use in Francisella tularensis. FEMS Immunol Med Microbiol 13:257-60.

4. Omsland A, Beare PA, Hill J, Cockrell DC, Howe D, Hansen B, Samuel JE, Heinzen RA. 2011. Isolation from animal tissue and genetic transformation of Coxiella burnetii are facilitated by an improved axenic growth medium. Appl Environ Microbiol 77:3720-5.

5. Beare PA, Jeffrey BM, Long CM, Martens CM, Heinzen RA. 2018. Genetic mechanisms of Coxiella burnetii lipopolysaccharide phase variation. PLoS Pathog 14:e1006922.

6. Anderson J. 2006. Part:BBa_J23100. <https://parts.igem.org/Part:BBa_J23100>. Accessed 01/03/2025.

7. ATUM(DNA2.0). ProteinPaintbox®. <https://www.atum.bio/catalog/reagents/protein-paint-box#__protein-overview>. Accessed 1/20.

8. Okunishi I, Kawagishi I, Homma M. 1996. Cloning and characterization of motY, a gene coding for a component of the sodium-driven flagellar motor in Vibrio alginolyticus. J Bacteriol 178:2409-15.

9. Gallagher LA, McKevitt M, Ramage ER, Manoil C. 2008. Genetic dissection of the Francisella novicida restriction barrier. J Bacteriol 190:7830-7.

10. Cocioba SS. Rainbow Chromoprotein Plasmid Set. <https://atinygreencell.com/products/rainbow-chromoprotein-expression-plasmid-set>. Accessed 1/12/2025.

11. Rodriguez SA, Davis G, Klose KE. 2009. Targeted gene disruption in Francisella tularensis by group II introns. Methods 49:270-4.
